# Effect of Singlet Oxygen on the Stomatal and Cell Wall of Rice Seedling Under Different Stresses

**DOI:** 10.3390/ijms26178382

**Published:** 2025-08-28

**Authors:** Yao Xiao, Zhong-Wei Zhang, Xin-Yue Yang, Lin-Bei Xie, Li-Ping Chen, Yang-Er Chen, Ming Yuan, Guang-Deng Chen, Shu Yuan

**Affiliations:** 1 International Science and Technology Cooperation Base for Efficient Utilization of Nutrient Resources and Fertilizer Innovation, College of Resources, Sichuan Agricultural University, Chengdu 611130, China; xiaoyao20230402@163.com (Y.X.); zzwzhang@126.com (Z.-W.Z.); yang16970319@163.com (X.-Y.Y.); kkskado@163.com (L.-B.X.); 18215892150@163.com (L.-P.C.); gdchen@sicau.edu.cn (G.-D.C.); 2 College of Life Science, Sichuan Agricultural University, Ya’an 625014, China; anty9826@163.com (Y.-E.C.); yuanming@sicau.edu.cn (M.Y.)

**Keywords:** singlet oxygen, Rose Bengal, stomatal density, cell wall, rice

## Abstract

Singlet oxygen (^1^O_2_), a reactive oxygen species, can oxidize lipids, proteins, and DNA at high concentrations, leading to cell death. Despite its extremely short half-life (10^−5^ s), ^1^O_2_ acts as a critical signaling molecule, triggering a retrograde pathway from chloroplasts to the nucleus to regulate nuclear gene expression. In this study, rice seeds were treated with 0, 5, 20 and 80 μM Rose Bengal (RB, a photosensitizer) under moderate light for 3 days to induce ^1^O_2_ generation. Treatment with 20 μM RB reduced stomatal density by approximately 25% in three-leaf-stage rice seedlings, while increasing the contents of pectin, hemicellulose, and cellulose in root cell walls by 30–40%. Under drought, salinity, or shading stress, 20 μM RB treatment significantly improved rice tolerance, as evidenced by higher relative water contents (49–58%) and chlorophyll contents (60–76%) and lower malondialdehyde (37–43%) and electrolyte leakage (29–37%) compared to the control. Moreover, RT-qPCR analysis revealed that the significant up-regulation of stomatal development genes (*OsTMM* and *OsβCA1*) and cell wall biosynthesis genes (*OsF8H* and *OsLRX2*) was associated with RB-induced ^1^O_2_ production. Thus, under controlled environmental conditions, ^1^O_2_ may regulate stomatal development and cell wall remodeling to enhance rice tolerance to multiple abiotic stresses. These results provide new perspectives for the improvement of rice stress tolerance.

## 1. Introduction

Reactive oxygen species (ROS), by-products of various metabolic pathways in plants, comprise hydrogen peroxide (H_2_O_2_), singlet oxygen (^1^O_2_), superoxide anion radicals (O_2_^−^), and hydroxyl radicals (OH^.^). These molecules regulate diverse physiological processes in plants [1]. Under normal conditions, enzymatic ROS-scavenging enzymes and non-enzymatic mechanisms(e.g., antioxidants) maintain ROS homeostasis [1,2]. However, biotic and abiotic stresses—particularly drought, salinity, and high light—disrupt the ROS production-scavenging equilibrium [3,4,5]. ROS at high level causes oxidative damage to lipids, proteins, and nucleic acids. In contrast, ROS at moderate level act as signaling molecules that modulate stomatal development, programmed cell death (PCD), and pathogen defense [6,7].

When plants encounter biotic or abiotic stresses, significant amounts of ^1^O_2_ are produced in both photosynthetic and non-photosynthetic tissues [8]. Despite its brief half-life (10^−5^ s), ^1^O_2_ can diffuse across cell membranes [8,9]. During high-light stress, for instance, ^1^O_2_ generated in *Chlamydomonas reinhardtii* photosystem II (PSII) diffuses through the cell membrane to induce expression of the glutathione reductase homolog GPXH [10]. ^1^O_2_ has also been detected in the roots of *Arabidopsis* under osmotic and drought stress [11], demonstrating its production in different non-photosynthetic compartments.

Under severe environmental stress, high concentrations of ^1^O_2_ damage cellular components through photoinhibition and uncontrolled cell death, impairing normal plant function [12]. It has been reported that more than 80% of nonenzymatic lipid peroxidation in plant cells is caused by ^1^O_2_ [3]. However, at sub-lethal concentrations, ^1^O_2_ initiates signaling pathways activating two distinct responses: one mediating high-light acclimation by redox homeostasis and another regulating PCD by regulating gene expression profiles [6,12,13].

The *Arabidopsis thaliana flu* mutant is one of the earliest identified plants capable of generating ^1^O_2_ specifically [14]. The FLUORESCENT (FLU) protein, a nuclear-encoded plastid protein, negatively regulates chlorophyll biosynthesis [14]. Loss of *FLU* function causes substantial accumulation of free protochlorophyllide (Pchlide), which efficiently produces ^1^O_2_ upon light exposure [14,15]. Consequently, when *flu* seedlings are transferred from darkness to light under alternating light/dark cycles, their cotyledons undergo chlorophyll degradation and rapid cell death. However, under continuous light, *flu* seedlings show no phenotypic difference from wild-type plants [16]. This indicates that ^1^O_2_ is produced in *flu* plastids in a controllable and non-invasive manner. Critically, *flu* seedling mortality and growth arrest result not from ^1^O_2_-mediated oxidative damage, but from activation of stress-responsive genetic programs [3,16]. Notably, during early stress responses, ^1^O_2_-induced genes regulate PCD via mechanisms distinct from those activated by O_2_^−^ and H_2_O_2_ [13].

In addition to free Pchlide molecules, artificial photosensitizers like Rose Bengal (RB) were widely used in ^1^O_2_ signaling research [17,18,19]. Koh et al. [17] demonstrated that RB localizes to the plasma membrane. It has photosensitizing activity and induces cell death, albeit through distinct mechanisms, suggesting ^1^O_2_ activates multiple cell death pathways. Li et al. [19] further showed that RB induces ^1^O_2_ production, binds the ^1^O_2_ receptor EXECUTER1 (EX1), and facilitates EX1 translocation from chloroplasts to the nucleus. There, EX1 interacts with transcription factors WRKY18 and WRKY40, forming a transcriptional complex that co-regulates nuclear gene expression. Thus, RB can be used as a validated chemical genetics tool for generating ^1^O_2_ signaling in plants.

Our previous study demonstrated that ^1^O_2_ signaling modulates nuclear gene expression and significantly enhances *Arabidopsis* tolerance to drought, salinity, and high-light stress by increasing cell wall thickness and reducing transpiration rates [20]. ^1^O_2_ production can be induced either by *FLU* gene deletion or RB treatment, which enhances stress tolerance under optimized light conditions without inhibiting plant growth. Despite these advances, the subcellular sites where ^1^O_2_ signaling is perceived and the mechanisms transmitting it to the nucleus remain poorly characterized, with no new nuclear signaling components identified [21]. Current ^1^O_2_ research focuses primarily on *Arabidopsis*.

Rice, a vital global food crop, has received considerable attention for research into its adaptability under abiotic stresses like drought, high temperature, and salinity. Functioning as a primary channel for plant-environment communication, stomata play a crucial role in plant development and function regulation in response to such stresses [22,23]. Recent studies indicated that reducing stomatal density can enhance rice’s abiotic stress tolerance [24,25]. Cell wall, a critical structural component of plant cells, significantly influences plant growth, morphology, and function. Plants respond to cell wall damage and enhance their adaptability and stress resistance by regulating cell wall synthesis and repair [26]. Recent research by Jia et al. [27] demonstrated that modulating cell wall biosynthesis can effectively enhance drought tolerance in rice. However, it remains unclear whether ^1^O_2_ is involved in stomatal development, cell wall biosynthesis, and abiotic stress tolerance in rice.

In this report, we investigated how RB, a singlet oxygen inducer, affects the growth and development of rice by regulating stomatal density and cell walls. In addition, we studied the mechanism of action of ^1^O_2_ as a signaling molecule in rice responses to abiotic stresses such as drought, salt, and shading. These findings lay the foundation for the improvement of rice stress tolerance.

## 2. Results

### 2.1. Exogenous RB Treatment Promotes the Production of Singlet Oxygen in Rice

**Figure 1:**
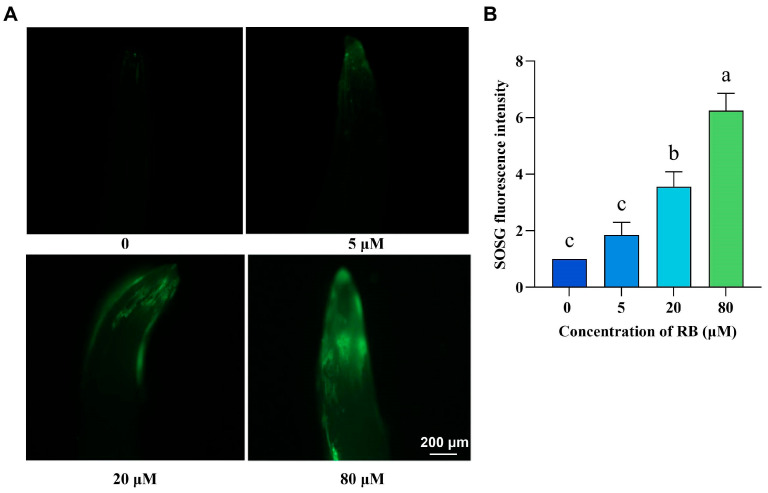
^1^O_2_ levels of rice seeds under different RB concentration treatments. (**A**) Representative images of rice seeds stained with SOSG solution under various RB concentration treatments; Bar = 200 μm; ^1^O_2_ fluorescence signal quantification results (**B**), with the ^1^O_2_ fluorescence signal expression level of control normalized to 1; 0, 5, 20, and 80 μM represent the concentrations of RB. Error bars in the graphs indicate the mean ± standard deviation (SD) of ten biological replicates from independent rice plants, and different lowercase letters denote significant differences at the 0.05 (*p* < 0.05) level.

Rose Bengal (RB), a photosensitizer that transfers energy to oxygen, generates ^1^O_2_ [17,18]. To determine whether exogenous RB induces ^1^O_2_ accumulation in rice, we treated seeds with varying RB concentrations and quantified ^1^O_2_ levels using Singlet Oxygen Sensor Green (SOSG). SOSG exhibits high selectivity for ^1^O_2_ through physical quenching, showing minimal reactivity with other ROS such as O_2_^−^,OH^.^, and NO [28]. As depicted in Figure 1, following RB treatment, the sprouts of rice seeds displayed a marked accumulation of green fluorescent signals (Figure 1A). Quantitative analysis revealed no significant difference in ^1^O_2_ fluorescence intensity between 5 μM RB treatment and controls. However, ^1^O_2_ fluorescence intensity increased significantly at both 20 μM and 80 μM RB treatments compared to controls (Figure 1B). These results demonstrate that exogenous RB effectively induces ^1^O_2_ accumulation in rice.

### 2.2. Singlet Oxygen Induces Stomatal Density Reduction and Cell Wall Thickening in Rice Seedlings

**Figure 2:**
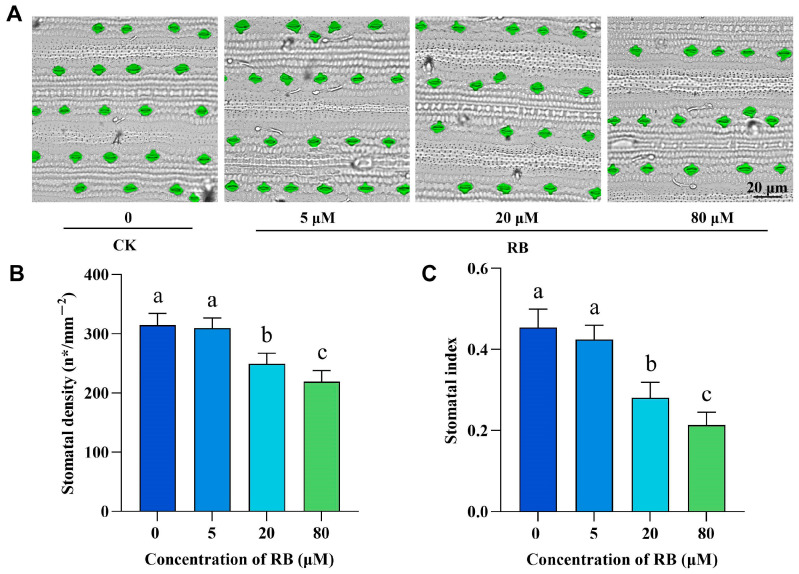
The effect of RB on stomatal traits in rice seedlings. (**A**) Stomatal images of 14-day rice seedling leaves under a microscope, with stomata pseudocolored in green for ease of identification. Bar = 20 μm. Statistics were obtained for (**B**) stomatal density and stomatal index (**C**). CK, control; 5, 20, and 80 μM represent the concentrations of RB. Error bars in the plots indicate the mean ± SD of ten biological replicates from independent rice plants, and different lowercase letters indicate significant differences at the 0.05 (*p* < 0.05) level.

Stomata regulate plant-atmosphere gas exchange, with their density and patterning critically influencing environmental adaptation [29,30]. To assess exogenous RB’s effects on rice stomatal development, we transferred the rice treated with RB to Kimura B nutrient solution and grew it for 14 days. Microscopic observation revealed intact stomata (Figure 2), showing progressively reduced stomatal density with increasing RB concentrations (Figure 2A). Subsequently, we quantified the stomatal density and stomatal index (Figure 2B,C). Results indicated no difference in stomatal density or index between rice treated with 5 μM RB and controls. At 20 μM RB, stomatal density decreased by 25% relative to controls, with a more pronounced reduction in stomatal index (35%). Under 80 μM RB treatment, stomatal density declined by approximately 30%, whereas stomatal index decreased substantially by 50% compared to controls.

**Figure 3:**
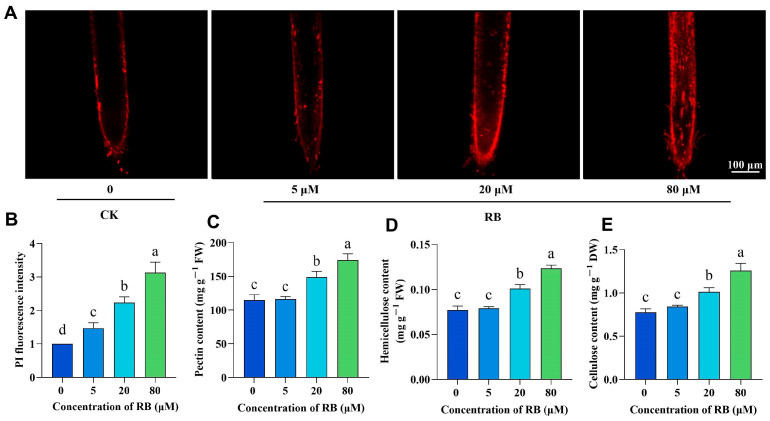
The effect of RB on the cell wall of rice roots. (**A**) Propidium iodide (PI) staining of cell walls from 14-day-old rice roots. Bar = 100 μm. Quantification of PI fluorescence signals (**B**), pectin (**C**), hemicellulose (**D**), and cellulose (**E**) content in cell wall fractions of rice roots. CK, control; 5, 20, and 80 μM represent the concentrations of RB; PI fluorescence signals are expressed as a percentage of CK levels, which were normalized to 1. FW, fresh weight. DW, dry weight. Error bars in the graphs indicate the mean ± SD of three biological replicates, and different lowercase letters denote significant differences at the 0.05 (*p* < 0.05) level.

Cell walls mediate resistance to biotic and abiotic stresses [31]. To assess root cell wall modifications, rice root tips were stained with propidium iodide (PI) across RB concentrations. This enabled visualization of structural changes (Figure 3), revealing progressively intensified PI fluorescence in root tips with increasing RB treatment (Figure 3A). Quantitative analysis revealed a positive correlation between RB concentration and PI fluorescence intensity (Figure 3B). Although 5 μM RB elicited no significant change in cell wall components versus controls, both 20 μM and 80 μM treatments significantly increased pectin, cellulose, and hemicellulose contents by 30–40% (Figure 3C–E). Thus, these results indicate that moderate ^1^O_2_ signaling reduces stomatal density while promoting cell wall component accumulation in rice seedlings.

### 2.3. Moderate Singlet Oxygen Signals Enhance Rice Tolerance to Abiotic Stress

**Figure 4:**
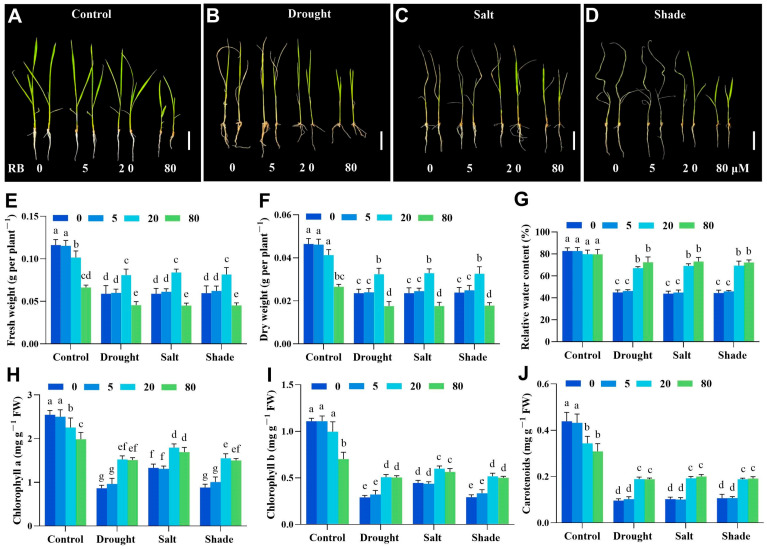
Moderate ^1^O_2_ enhanced rice tolerance to drought, salinity, and shade stress. The phenotype changes in three-week-old rice seedlings under no stress (**A**), drought stress (**B**), salt stress (**C**), and shade stress (**D**) were observed after 14 days. Bar = 5 cm. The fresh weight (**E**), dry weight (**F**), and moisture content (**G**), as well as the chlorophyll a, chlorophyll b, and carotenoid content (**H**–**J**) of the rice seedlings are also presented. 0, 5, 20, and 80 μM represent the concentrations of RB. FW, fresh weight. The error bars in the graph indicate the mean of three biological replicates ± SD, and different lowercase letters denote significant differences at the 0.05 (*p* < 0.05) level.

To assess ^1^O_2_-mediated protection against abiotic stress, we subjected three-leaf-stage rice seedlings to a 14-day simulation of drought, salt, and shading stresses. Under all stress conditions, untreated seedlings exhibited severe leaf curling and wilting relative to unstressed controls. Although biomass did not differ significantly between rice treated with 5 μM RB and untreated controls, both 20 μM and 80 μM RB treatments significantly mitigated biomass reduction (Figure 4A–D). Under non-stress conditions, 5 μM RB treatment showed no significant effect on rice biomass. While 20 μM RB caused a minor biomass reduction (<10%), and 80 μM RB treatment significantly decreased biomass by approximately 43% (Figure 4E,F). Additionally, seedlings exposed to 20 μM or 80 μM RB exhibited higher relative water contents (49–62%) compared to controls (Figure 4G).

Stress conditions also reduced photosynthetic pigment content (chlorophyll a, chlorophyll b, and carotenoids) versus controls. Crucially, 20 μM and 80 μM RB treatments significantly maintained pigment levels compared to the absence of RB treatment. Conversely, under non-stress conditions, 5 μM RB showed no effect on chlorophyll levels, 20 μM RB reduced chlorophyll content by 11%, and 80 μM RB significantly decreased chlorophyll by approximately 37% versus controls (Figure 4H–J). These results demonstrate that moderate ^1^O_2_ enhances rice tolerance to drought, salinity, and shade stress through coordinated physiological preservation.

### 2.4. Moderate Singlet Oxygen Alleviates Oxidative Damage in Rice Under Different Stresses

**Figure 5:**
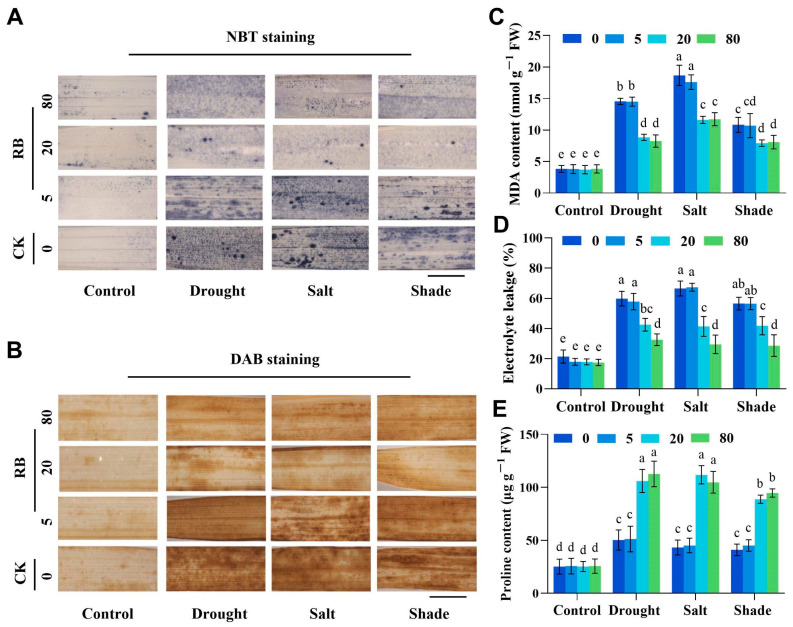
Oxidative damage parameters of rice seedlings under three stress conditions. (**A**) O_2_^−^ and (**B**) H_2_O_2_ staining in leaves. Bar = 5 cm. Malondialdehyde content (**C**), relative electrical conductivity (**D**), and Proline content (**E**). CK, control; 5, 20, and 80 μM represent the concentrations of RB. FW, fresh weight. Error bars in the graphs indicate the mean ± SD of three biological replicates, and different lowercase letters denote significant differences at the 0.05 (*p* < 0.05) level.

Abiotic stresses, such as drought, salinity, and shading, can disrupt the redox balance in plants. To investigate whether RB affected the accumulation of ROS in rice seedlings under different stresses, nitroblue tetrazolium (NBT) and 3,3-diaminobenzidine (DAB) staining were used to assess ROS accumulation. We observed enhanced O_2_^−^ and H_2_O_2_ deposition in stressed leaves versus unstressed controls (Figure 5A,B). However, when concentrations of RB at 20 μM and 80 μM were used, the area and intensity of NBT and DAB staining in rice leaves under the three stresses were diminished compared to without RB treatment. Quantitative H_2_O_2_ and O_2_^−^ assays corroborated these findings (Figure S1A,B).

While RB concentrations showed no effect on MDA, electrolyte leakage (EL), or proline under control conditions, stress exposure markedly increased MDA and electrolyte leakage. Crucially, although no significant difference was observed between 5 μM RB-treated samples and untreated controls, both 20 μM and 80 μM RB treatments reduced MDA and EL for over 40% (Figure 5C,D). RB treatments at 20 μM and 80 μM significantly elevated proline contents in rice by 53–62% compared to controls (Figure 5E). In addition, antioxidant enzyme activities (SOD, POD, CAT, APX) remained unchanged by RB under control conditions but increased dose-dependently under stress (Figure S2A–D). Overall, the 20 μM RB mitigates abiotic stress-induced ROS accumulation and activates antioxidant systems, thereby alleviating oxidative damage in rice seedlings.

### 2.5. Singlet Oxygen Signaling Mediates Stomatal and Cell Wall Adaptations Under Abiotic Stresses

**Figure 6:**
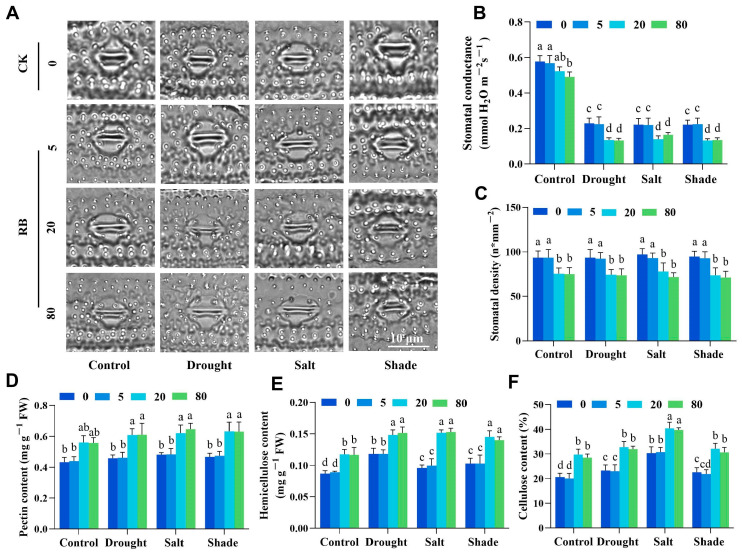
Alterations in stomatal characteristics and cell walls of rice seedlings subjected to drought, salt, and shade stresses. (**A**) Microscopic images of stomata on rice leaves under normal conditions and those exposed to drought, salt, and shade stresses. Bar = 10 μm. Stomatal conductance (**B**) and stomatal density (**C**) of rice seedlings. Contents of cell wall components, pectin (**D**), hemicellulose (**E**), and cellulose (**F**), in rice seedlings under normal conditions and each stress condition. Concentrations of 0, 5, 20, and 80 μM represent the RB levels. FW, fresh weight. Error bars in the graphs denote the mean ± SD of three biological replicates, with different lowercase letters indicating significant differences at the 0.05 (*p* < 0.05) level.

To determine whether ^1^O_2_ signaling confers rice tolerance to drought, salinity, and shade, we analyzed stomatal traits and cell wall composition under stress versus control conditions (Figure 6). Compared to no RB treatments, 20 and 80μM RB significantly reduced stomatal aperture (Figure 6A). Data analysis confirms no significant difference in stomatal density or index between 5 μM RB-treated plants and untreated controls under both stress and non-stress conditions. However, stomatal conductance decreased markedly under all stresses versus controls, particularly with 20 and 80 μM RB (Figure 6B). Treatments with 20 and 80 μM RB reduced stomatal density by 20–30% versus untreated controls; no significant density differences emerged among stress treatments (Figure 6C).

Additionally, we also measured the length, width, and pore diameter of the stomata, finding that stomatal width and pore diameter decreased dose-dependently with RB concentration (*p* < 0.05), unaffected by stress type (Figure S3A,B). However, stomatal length remained unchanged across treatments (Figure S3C).

Regarding alterations in the composition of rice root cell walls, the results revealed no significant difference between plants treated with 5 μM RB and untreated controls. Under all stress conditions, treatment with 20 μM RB significantly increased the contents of pectin, hemicellulose, and cellulose by 25–41%, whereas 80 μM RB treatment resulted in increases of 29–43%. However, no significant variation in cell wall compositions was observed across different stresses (Figure 6D–F). These findings demonstrate that ^1^O_2_ signaling reduces stomatal density and aperture while enhancing cell wall component deposition to improve rice tolerance to drought, salinity, and low-light stress.

### 2.6. Singlet Oxygen Regulates Stomatal Development and Cell Wall Synthesis Genes to Enhance Abiotic Stress Tolerance

**Figure 7:**
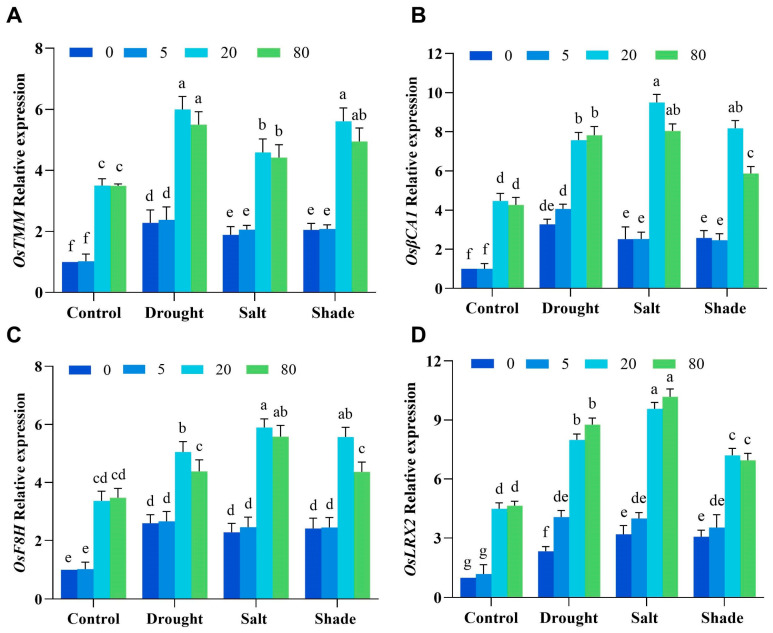
The effects of RB on genes associated with stomatal development and cell wall synthesis in rice under various stress conditions were examined. Quantitative real-time PCR analysis was employed to assess the expression levels of representative genes *OsTMM* (**A**), *OsβCA1* (**B**), *OsF8H* (**C**), and *OsLRX2* (**D**). The expression levels of the control seedlings were normalized to 1. Error bars in the graphs represent the mean ± SD of three biological replicates, with different lowercase letters denoting significant differences at the 0.05 (*p* < 0.05) level.

To elucidate the molecular mechanism of ^1^O_2_ signaling in regulating stomatal density and cell wall synthesis in rice, we analyzed key gene expression under control and stress conditions (Figure 7). Under normal conditions, rice plants treated with 20 μM and 80 μM RB exhibited significantly elevated expression levels of key genes compared to untreated controls. Notably, under drought, salt, and shade stress conditions, these genes demonstrated substantially greater up-regulation relative to non-stressed conditions. These results demonstrate that optimal ^1^O_2_ signaling rapidly induces stomatal/cell wall regulatory genes, driving reduced stomatal density and enhanced cell wall component deposition to confer multi-stress tolerance in rice.

These genes include two involved in cell wall synthesis, *OsF8H* (fragile fiber 8 homolog) and *OsLRX2* (leucine-rich repeat/extensin 2); and two negative regulators associated with stomatal development, *OsβCA1* (β-carbonic anhydrase 1) and *OsTMM* (too many mouths). *F8H* encodes a key xylan biosynthesis regulator during secondary cell wall formation, functionally redundant with glycosyltransferase *FRA8* in glucuronoxylan (GX) synthesis [32]. *LRX2* produces a leucine-rich repeat/extensin protein critical for cell wall development and morphogenesis [33]. *TMM* encodes a leucine-rich repeat receptor-like kinase (LRR-RLK) that regulates stomatal patterning and in Arabidopsis [34], with rice homologs functionally characterized. *βCA1* encodes a β-carbonic anhydrase central to photosynthetic carbon assimilation, stomatal regulation, and immunity [35]. In rice, *OsβCA1* knockout impairs CO_2_ supply, reducing photosynthesis and disrupting stomatal closure [36].

## 3. Discussion

Reactive oxygen species (ROS) are an indispensable component of plants’ response to biotic and abiotic stresses. Moderate ROS levels function as signaling molecules that regulate plant stress responses by modulating stomatal closure, reinforcing root remodeling, and coordinating abscisic acid (ABA) and salicylic acid (SA) pathways [37,38,39,40]. Recent studies demonstrate that ^1^O_2_ enhances biotic stress resistance by inducing jasmonic acid biosynthesis and subsequent accumulation of defensive metabolites like sinigrin [41]. Besides inducing PCD, ^1^O_2_ signaling also participates in salicylic acid and jasmonic acid pathways to confer photosynthetic stress tolerance [42]. Furthermore, ^1^O_2_ signaling upregulates genes involved in oxidative stress response, hormone signaling, and detoxification via β-cyclocitral (β-CC) mediation, enhancing plant tolerance to high light stress [43]. Therefore, ^1^O_2_ appears to influence plants’ tolerance to biotic and abiotic stresses through different mechanisms.

In a previous study, we demonstrated that ^1^O_2_ upregulated stress response, cell wall biosynthesis, and stomatal developmental genes in *Arabidopsis thaliana*, reducing stomatal density and thickening cell walls [20]. In this study, we proved that two negative regulators of stomatal development, *OsTMM* and *OsβCA1*, as well as cell wall synthesis-related genes *OsF8H* and *OsLRX2*, were significantly upregulated by ^1^O_2_ in rice (Figure 7). However, the decrease in stomatal density in rice cannot be solely attributed to the high expression of *OsTMM* and *OsβCA1*. Cell wall dynamics, particularly guard cell pectin methyl esterification, critically regulate stomatal morphogenesis and environmental responsiveness [44]. Supporting this, *Arabidopsis sfr8* mutants with reduced cell wall fucose display altered stomatal complexes, suggesting fucosylation-dependent rhamnogalacturonan-II modifications may direct stomatal development [45].

Changes in the cell wall cannot be attributed solely to the high expression of *OsF8H* and *OsLRX2*. Salt, drought, and other osmotic stress treatments can lead to ROS accumulation and changes in the cell wall [46]. ROS accumulation can trigger cross-linking between phenolic resins and glycoproteins such as cell wall extensin, ultimately resulting in cell wall hardening [46]. A recent study showed that under osmotic stress conditions, ^1^O_2_-dependent lipoxygenase (LOX) activity locally bursts to mediate plastid remodeling, which further affects cell wall structure and function [47]. Thus, the detailed mechanism by which ^1^O_2_ signaling leads to changes in cell wall composition/structure and stomatal development requires further investigation.

Singlet oxygen signaling orchestrates reduced stomatal density and cell wall remodeling in plants—adaptations that enhance crop stress tolerance and potentially improve yield [21]. Stomatal reduction improves drought tolerance [48,49,50], salinity/osmotic resilience [51,52], and thermo-tolerance [53,54] through conserved water resources. Climate change intensifies abiotic stresses (water logging, high temperature, drought), creating urgent demand for water-efficient, high-yielding crops. Engineering drought-resilient stomatal architectures via developmental modulation represents a strategic solution [55]. Furthermore, reduced stomatal density may adversely affect plant photosynthetic performance. Zekri et al. [56] observed a significant correlation between decreased stomatal density and reduced photosynthetic rate under drought stress. However, under non-stress conditions, reduced stomatal density may not impair photosynthetic performance as significantly as it does under stress [22]. Additionally, regulating stomatal density could indirectly influence photosynthesis by altering stomatal conductance and intercellular CO_2_ concentration [57]. Therefore, the impact of singlet oxygen-induced stomatal density reduction on photosynthetic performance requires careful evaluation, considering different environmental conditions.

Changes in the cell wall greatly affect the stress resistance of plants. Cell walls provide the primary physical barrier against pathogens. Under biotic stress, plants fortify walls through compositional changes that inhibit infection [58,59]. Cell wall remodeling likewise enhances tolerance to drought [60,61], salt/osmotic stress [62,63], and waterlogging [64] by enhancing mechanical integrity and hydraulic conductivity. In addition, subtle cell wall defects caused by dysfunction of the putative pectin synthase AtCSLD5 in *Arabidopsis sos6* (*salt over-sensitive 6*) mutants led to oxidative stress and greatly increased sensitivity to osmotic and drought stress [65]. Strategic modification of wall composition/structure can thus simultaneously optimize stress resilience and biomass production [66]. Changes in cell wall components influence not only plant stress resistance but also the mechanical properties and growth of the plant itself. The mechanical characteristics of the cell wall, such as hardness and elasticity, determine cellular extensibility during growth [67]. Newly formed cell walls may exhibit greater rigidity than adjacent mature walls, potentially altering cell expansion and final organ morphology [67]. Furthermore, modifications to cell wall mechanics may regulate plant development by affecting the activity of cell wall-related enzymes [68]. Our study demonstrates that controlled RB application in rice development achieved both stomatal density reduction and cell wall component enhancement, significantly improving various stress tolerances.

Rose Bengal (RB) effectively inhibits vesicular glutamate transporter (VGlut) and vesicular monoamine transporter (VMAT) with Ki values of 19 and 64 nM, respectively. It exhibits low toxicity (minimum toxic dose: 437 mg/kg/day in female rats) and undergoes rapid photo-degradation [69]. Recent studies confirm RB acts as a photo oxidant to generate ^1^O_2_ signals [17,18,19]. We investigated the effects of different concentrations of RB on rice growth and development. No significant differences were observed between rice plants treated with 5 μM RB and those without RB addition. Rice seedlings treated with 20 μM RB exhibited slight growth inhibition, whereas those treated with 80 μM RB showed severe growth inhibition (Figure 4A), which may be due to toxicity at high concentrations. In summary, our study demonstrates that RB induces physiologically relevant ^1^O_2_ levels, reducing stomatal density and cell wall remodeling to confer different abiotic stress tolerance. This concentration (20 μM) is below the safety threshold and elicits no serious effects on rice growth.

Currently understanding of ^1^O_2_ signaling derives primarily from *Arabidopsis flu* mutants. Notably, rice contains two *FLU* homologs (*OsFLU1*, *OsFLU2*), and simultaneous knockout induces lethality. Li et al. [70] observed severe chlorosis in *OsFLU1* knockout seedlings, suggesting *FLU* genes may enhance environmental adaptability in crops. Exogenous RB enables non-transgenic ^1^O_2_ induction with precise spatiotemporal gene expression regulation, alleviating growth suppression from uncontrolled ^1^O_2_ accumulation. ^1^O_2_ signaling induced by RB may hold a broader value for breeding and agronomic applications. However, RB’s applicability across crop species and the optimal concentration for individual crops require further validation under practical conditions.

## 4. Materials and Methods

### 4.1. Plant Material and Growth Conditions

The experiment employedindica-type three-line hybrid rice *Oryza sativa* L. cv. Yixiangyou 2115. Seeds were surface-sterilized in 2% (*v*/*v*) sodium hypochlorite (Sigma-Aldrich, St. Louis, MO, USA) for 30 min, then germinated in gibberellin (Sigma-Aldrich) solution (4 mg L^−1^) until radicle emergence. Uniformly developed seeds were selected and soaked in Rose Bengal (RB; Sigma-Aldrich) solutions (0, 5, 20, or 80 μM) for 3 d. Lighting conditions during the RB soaking period consisted of 16-h light (120 µmol m^−2^ s^−1^) followed by 8-h dark cycles. Treated seeds were then transferred to modified Kimura B nutrient solution (0.5 mM (NH_4_)_2_SO_4_, 0.54 mM MgSO_4_·7H_2_O, 1 mM KNO_3_, 0.3 mM CaCl_2_, 0.18 mM KH_2_PO_4_, 0.09 mM K_2_SO_4_, 16 µM Na_2_SiO_3_·9H_2_O, 9.14 µM MnCl_2_·4H_2_O, 46.2 µM Na_2_MoO_4_·2H_2_O, 0.76 µM ZnSO_4_·7H_2_O, 0.32 µM CuSO_4_·5H_2_O, and 40 µM Fe(II)-EDTA, pH 5.8) and cultivated hydroponically under a controlled condition (21 ± 1 °C; 16/8-h light/dark photoperiod; 120 μmol·m^−2^·s^−1^) until the three-leaf stage (21 d).

For stress treatments, 21-day-old rice seedlings were transplanted into soil to simulate rice transplanting. Drought stress was simulated by withholding irrigation for 14 d. Salt stress refers to transplanting rice seedlings into soil containing 50 mM NaCl for 14 d. Shade stress was imposed by exposing transplanted seedlings to 35 μmol·m^−2^·s^−1^ irradiance for 14 d (21 ± 1 °C; 16/8-h light/dark).

### 4.2. Determination of Photosynthetic Pigment Content

Photosynthetic pigment content was determined following Lichtenthaler et al. [71]. Fresh rice leaves (0.2 g) were homogenized in 10 mL of 95% ethanol within a sealed centrifuge tube and incubated in darkness for 24 h. After complete tissue decolorization, 1 mL of supernatant was diluted with 5 mL of fresh 95% ethanol. Absorbance at 665, 649, and 470 nm was measured to quantify chlorophyll a (Chl a), chlorophyll b (Chl b), and carotenoid content, respectively.

### 4.3. Stomatal Count

Rice leaf stomatal traits were observed using the method described by Kusumiet al. [72]. The upper epidermis of the leaf from the second fully expanded leaf, located near the tip within a 3–5 cm area, was selected, fixed, and coated with a layer of transparent nail polish (Wanke Scientific Comp., Chengdu, China). The leaf blade was then allowed to dry naturally for 5 min. Subsequently, the leaf blade was detached from the plant and affixed with the nail polish side facing transparent adhesive tape, and the stomata on the leaf blade were mapped onto the nail polish. A transparent clinical slide was then placed under an inverted fluorescence microscope (Axio Imager Microscope 2, Chengdu, China) and photographed. The stomatal density, length, width, area, and aperture were calculated using Image J software version 1.53 [73].

### 4.4. Microscopy Analysis of Root Cell Walls

To observe the changes in the cell walls of rice roots, we utilized the propidium iodide (PI; Sigma-Aldrich) staining method [74]. Initially, the roots of 14-day-old rice plants were selected, thoroughly rinsed, and subsequently stained with 30 μg/mL PI for 30 min at room temperature, ensuring they were shielded from light. The fluorescent signals from the roots were then captured using excitation light at a wavelength of 595 nm, and images were taken with a Leica SP2 laser confocal microscope (Leica, Buffalo Grove, IL, USA).

### 4.5. Pectin and Cellulose Content Determination

Following the protocols of Xiao et al. [75] and Du et al. [76], cell wall fractions were obtained from rice roots after 14 d of growth and following stress. Pectin was extracted from the cell wall by mixing 2 mg of the extract with 1 mL of deionized water and heating it in a boiling water bath for 1 h. The mixture was then rapidly cooled and centrifuged at 4500× *g* for 10 min. This process was repeated three times, and the supernatant from each collection was combined to form the pectin extract. The content of furfural was determined using galacturonic acid (Sigma-Aldrich) as a reference.

### 4.6. ROS Staining and Quantification of Oxidative Damages

^1^O_2_ levels were determined using a single-linear oxygen green fluorescent probe (SOSG) [77]. Rice seeds treated with varying concentrations of RB were stained in a 20 μM SOSG (Thermo Fisher Scientific, St. Louis, MO, USA) solution for 20 min in the dark, and imaged under the inverted fluorescence microscope. Excitation of SOSG occurred at 488 nm, with fluorescence recorded across the spectral range of 510–600 nm.

Regarding the accumulation of superoxide anions (O_2_^−^) and hydrogen peroxide (H_2_O_2_) in rice leaves, staining with (nitroblue tetrazolium) NBT (Sigma-Aldrich) and (3,3-diaminobenzidine) DAB (Sigma-Aldrich) was first used for observation [78]. Rice leaves were cut and stained with 0.8 mg/mL NBT or 2.4 mg/mL DAB for 3 h. The samples were then destained in 75% ethanol and photographed and preserved using a stereomicroscope (Leica Microsystems M 165 C/FC). Quantitative determination of H_2_O_2_ and O_2_^−^ content in rice leaves, as described previously [79]. Rice leaves (0.5 g) were collected 14 d after stress treatment, grounded in liquid nitrogen, and homogenized in 5 mL 0.1% (*w*/*v*) trichloroacetic acid (TCA; Sigma-Aldrich). The homogenate was centrifuged at 12,000× *g* for 20 min at 4 °C. The supernatant (1 mL) was mixed with 10 mM sodium phosphate buffer (0.5 mL, pH 7.0) and 1 M KI (1 mL). Absorbance was measured at 390 nm to quantify H_2_O_2_ content. For O_2_^−^ content, 0.5 g leaf tissue was powdered in 65 mM sodium phosphate buffer (1.5 mL, pH 7.8) and centrifuged at 10,000× *g* for 15 min. The supernatant (0.5 mL) was combined with 0.5 mL sodium phosphate buffer and 10 mM hydroxylamine hydrochloride (0.1 mL). After incubation at 25 °C for 20 min, 58 mM p-aminobenzenesulfonic acid (1 mL) and 7 mM α-naphthylamine (1 mL) were added, followed by another 20 min incubation at 25 °C. The final mixture was extracted with an equal volume of chloroform, and the absorbance of the aqueous phase was measured at 530 nm to quantify the O_2_^−^ content.

The content of Malondialdehyde (MDA) was determined using the thiobarbituric acid-reactive substances (TBARS; Sigma-Aldrich) method [80]. Electrolyte leakage was measured using Dionisio-Sese and Tobita’s method [81], and relative water content was determined according to Arndt et al. [82].

### 4.7. Determination of Antioxidant Enzyme Activities

Superoxide dismutase (SOD) measurements were determined following the protocol of Liu et al. [83]. Peroxidase (POD) activity was assessed according to the method described by Verma and Mishra [84]. Catalase (CAT) activity and ascorbate peroxidase (APX) measurements were conducted using the procedures outlined by Esfandiari et al. [85].

### 4.8. Quantitative Real-Time PCR Analysis

*OsTMM* (*Too Many Mouths*), *OsβCA1* (*β-Carbonic Anhydrase 1*), *OsF8H* (*Fragile Fiber 8 Homolog*), and *OsLRX2* (*Leucine-Rich Repeat*/*Extensin 2*) expression levels were analyzed by quantitative RT-PCR (qPCR) using SYBR Premix Ex Taq (Takara Biomedical Technology, Dalian, China). The threshold cycle (Ct) value—defined as the PCR cycle where reporter fluorescence exceeds background levels—determines initial target gene copy numbers [86]. Three biological replicates were conducted for each sample. The *OsACTIN1* gene served as an internal control. The expression level of rice seedlings without RB and stress treatment was normalized to 1. All primers are listed in Supplementary Table S1.

### 4.9. Data Analysis

Statistical analysis was performed using GraphPad Prism 10 (Free Image public license version 1.0) for graphical representation. All experiments were performed at least three times, and mean values are presented with standard deviations (*n* ≥ 3). Statistical analysis was performed based on analysis of variance (ANOVA) using Tukey’s multiple comparisons test. The differences were judged statistically significant at *p* < 0.05.

## 5. Conclusions

The photosensitizer RB can induce ^1^O_2_ production in rice. Treatment with 20 μM RB-mediated ^1^O_2_ led to reduced stomatal density and enhanced cell wall thickening. Under drought, salt, or shade stress, 20 μM RB treatment significantly decreased MDA and EL levels in rice leaves, while elevating the activities of SOD, POD, and APX, thereby enhancing abiotic stress tolerance in rice seedlings. Additionally, the significant up-regulation of key negative regulators of stomatal development (*OsTMM* and *OsβCA1*) and cell wall-related genes (*OsF8H* and *OsLRX2*) was correlated with RB-induced ^1^O_2_ production. Thus, singlet oxygen likely enhances rice resilience to diverse abiotic stresses by modulating stomatal development and cell wall remodeling. These findings provide a theoretical foundation for improving crop stress resistance.

## Data Availability

All data generated or analyzed during this study are included in this published article and its Supplementary Information file.

## Supplementary Materials

The following supporting information can be downloaded at: https://www.mdpi.com/article/10.3390/ijms26178382/s1.
