# Supplementary material for: Effect of Singlet Oxygen on the Stomatal and Cell Wall of Rice Seedling Under Different Stresses
Source: Int J Mol Sci. 2025 Aug 28;26(17):8382. doi: 10.3390/ijms26178382 (PMC12428634; doi:10.3390/ijms26178382)
Supplement: Supplementary file 1 [file ijms-26-08382-s001.zip › ijms-3820782-supplementary.pdf]

Supplemental Table S1. Primers used for RT-qPCR.

Gene name	Forward primer	Reverse primer	Size (bp)
OsTMM	5'-ACCTCACATCCGCCATCCC-3'	5'-GCCGCCACATCATCTGCTT-3'	621
OsβCA1	5'-ATACTCACAGCGAAATCAATAC-3'	5'-GGCTCCCATAAGTCCAAG-3'	643
OsF8H	5'-GGCTCGCTCCTTGTTCTGC-3'	5'-AAGGTCCTTGCTATGGATGGTGT-3'	530
OsLRX2	5'-GCTCAGGAAGGTGACGGTGTT-3'	5'-GGCGGCATTGAGGATTTGG-3'	513
OsActin1	5'-TAGGAGGAAATGGCTGACGG-3'	5'-CAAGGGCCACATATGCAAGC-3'	679

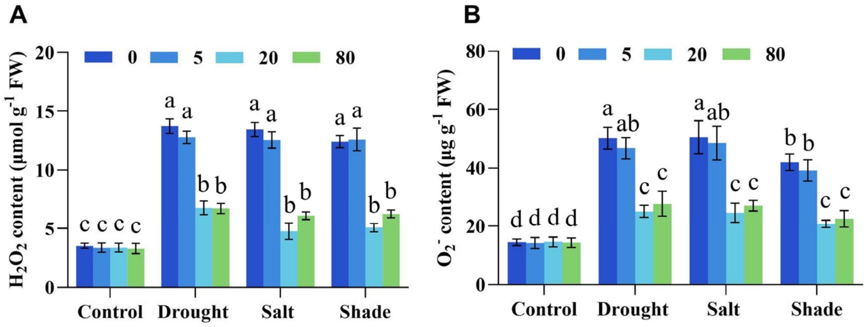

Supplementary Figure S1. Levels of hydrogen peroxide (H_2O_2) and superoxide anions ($O_2^{\bullet-}$) in rice leaves under normal conditions versus three types of stress. Error bars in the graphs indicate the mean ± SD of three biological replicates, and different lowercase letters denote significant differences at the 0.05 ($p < 0.05$) level.

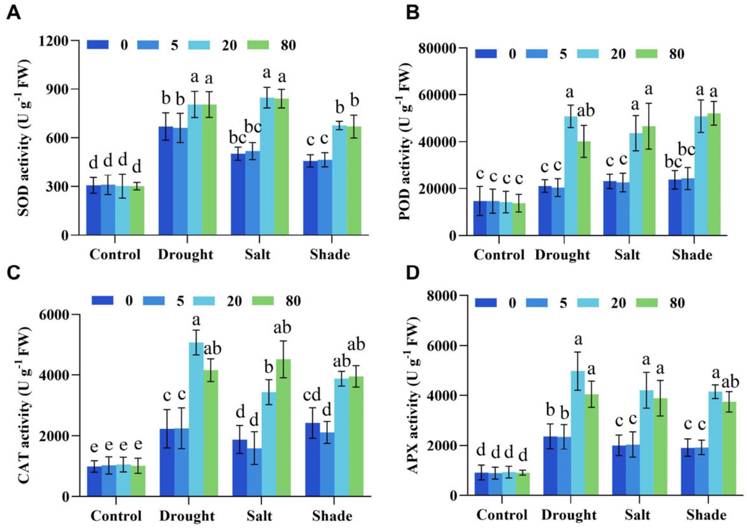

Supplementary Figure S2. Measurement of the activity of relevant enzymes in rice seedlings under normal conditions and three types of stress. Error bars in the graphs indicate the mean ± SD of three biological replicates, and different lowercase letters denote significant differences at the 0.05 ($p < 0.05$) level.

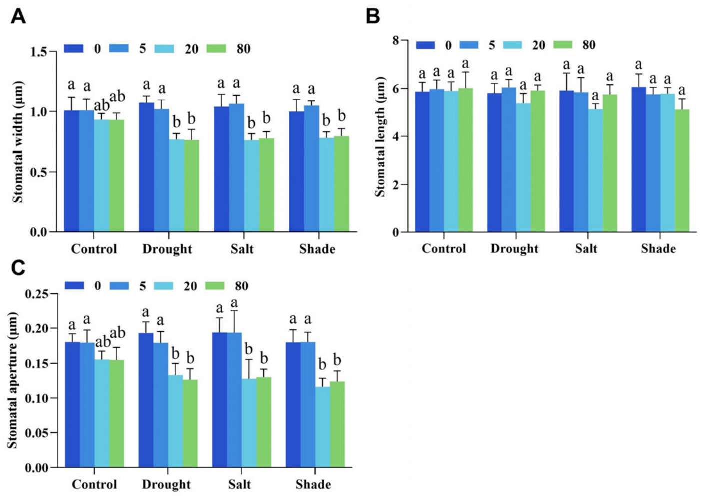

Supplementary Figure S3. Stomatal characteristics of rice leaves in normal conditions versus under three types of stress. Error bars in the graphs indicate the mean ± SD of three biological replicates, and different lowercase letters denote significant differences at the 0.05 (p < 0.05) level.
